# Identification, discrimination and heterogeneity of fibroblasts

**DOI:** 10.1038/s41467-022-30633-9

**Published:** 2022-06-14

**Authors:** Urban Lendahl, Lars Muhl, Christer Betsholtz

**Affiliations:** 1 Department of Cell and Molecular Biology, Karolinska Institutet, SE-171 77 Stockholm, Sweden; 2 Department of Neurobiology, Care sciences and Society, Karolinska Institutet, SE-14183 Huddinge, Sweden; 3 Department of Medicine, Huddinge, Karolinska Institutet, Blickagången 16, SE-141 57 Huddinge, Sweden; 4 Department of Immunology, Genetics and Pathology, Rudbeck Laboratory, Uppsala University, Dag Hammarskjölds väg 20, SE-751 85 Uppsala, Sweden

**Keywords:** Mechanisms of disease, Cells

## Abstract

Fibroblasts, the principal cell type of connective tissue, secrete extracellular matrix components during tissue development, homeostasis, repair and disease. Despite this crucial role, the identification and distinction of fibroblasts from other cell types are challenging and laden with caveats. Rapid progress in single-cell transcriptomics now yields detailed molecular portraits of fibroblasts and other cell types in our bodies, which complement and enrich classical histological and immunological descriptions, improve cell class definitions and guide further studies on the functional heterogeneity of cell subtypes and states, origins and fates in physiological and pathological processes. In this review, we summarize and discuss recent advances in the understanding of fibroblast identification and heterogeneity and how they discriminate from other cell types.

## Abstract (web summary)

In this review, the authors look at how recent progress in single-cell transcriptomics complement and enrich the classical, largely morphological, portraits of fibroblasts. The detailed molecular information now available provides new insights into fibroblast identity, heterogeneity and function.

## Introduction

Fibroblasts were first described in 1858 by Rudolf Virchow as cells located in connective tissue^1^, a term coined already in 1830 by Johannes Peter Müller for the matter that connects and mechanically supports other tissues^2^. In 1879, Matthias Duvall distinguished fibroblasts from epithelial cells within the mesenchyme of chick embryos, thereby adding to their demarcation^3^. Progressively, it became clear that fibroblasts reside within all dense and loose fibrous connective tissues. Dense fibrous connective tissue makes up tendons, ligaments and fasciae of the musculoskeletal system. Loose fibrous connective tissue is located at many sites, including visceral organ capsules, beneath the epithelial linings of airways, gastrointestinal and urogenital tracts (laminae propria) and skin (dermis), in central nervous system meninges, and between parenchymal cells in muscle and adipose tissue. Fibroblasts are thus a major cellular component of the diverse fibrous connective tissues that disperse throughout our organs^2,4^. Alongside endothelial and blood cells, fibroblasts are one of the most common and widespread cell types in our bodies^2^.

The term *fibroblast*, used already more than 100 years ago by Ernst Ziegler^5^ and Alexander Maximow^6^, deserves a comment, as the “-blast” suffix refers to morphological hallmarks of active protein synthesis. A key research goal of the early 20th century was to identify the cellular source of extracellular matrix (ECM) during tissue repair^4,7–9^. In these studies, the quiescent fibroblasts of uninjured organs were sometimes referred to as *fibrocytes*, which displayed a small cytoplasm, few ribosomes and condensed chromatin, together suggesting low levels of protein synthesis^10^. It was assumed that fibrocytes give rise to fibroblasts upon wounding, and conversely that fibroblasts differentiate into fibrocytes during healing^10^. Perhaps due to limited interest in the quiescent cells, the original meaning of fibrocyte was progressively lost and *fibroblast* adopted as the common name irrespective of its state of activity. *Fibrocyte* later reappeared as the name for a circulating monocytes/macrophages-related antigen-presenting cell involved in tissue repair^11,12^ (for review see ref. ^13^).

In the early days of cell culturing, fibroblasts turned out to be sturdy and easy to propagate on artificial surfaces such as glass and plastic. They regularly outgrew other cell types and thus became popular for basic studies in cell biology. In the 1950s, Michael Abercrombie and Joan Heaysman established the important concept of cell contact inhibition using fibroblasts^14^, and Leonard Hayflick showed in 1965 that fibroblasts stop dividing and senesce after a finite number of population doublings in vitro^15^, thereby disproving the view that cultured cells could be passaged indefinitely (see ref. ^16^). Fibroblasts were further utilized to identify cellular oncogenes in the 1970s and 1980s^17^, as well as for the more recent establishment of techniques for induced pluripotency^18^. Fibroblasts are also commonly used as feeder cells in co-culture experiments to provide cell contacts, ECM and growth factors needed for other cells, such as embryonic stem cells, to thrive^19,20^.

It is well-established that fibroblasts produce collagens and numerous other proteins that make up the ECM of fibrous connective tissues. In fact, one of the fibroblasts’ archetypical products, collagen type 1, is the most abundant protein in our bodies^21^. The ECM consists of more than 300 different core proteins and numerous additional bridging proteins and matrix-modifying enzymes, together referred to as the matrisome^22^. Each tissue has its unique ECM composition, and it was early recognized, based both on morphological and molecular criteria, including gene expression profiling, that fibroblasts from various anatomical locations differ^23,24^. In addition to ECM production, fibroblasts play a role in wound contraction. In 1971, Giulio Gabbiani and colleagues reported that fibroblasts respond to tissue injury by assuming a contractile, *myofibroblast*, phenotype^25^. Similar cells are implicated in fibrosis, which may be defined as unresolved repair after tissue damage (for review, see ref. ^26^). Besides fibrosis, fibroblasts are important also for other disease processes, including cancer where they make up a variable proportion of the tumor stroma and deposit ECM of various molecular compositions and secrete different cocktails of growth factors and cytokines that influence tumor growth and metastasis^27,28^. Secretion of soluble extracellular signaling molecules is also of profound physiological importance during development, where fibroblasts provide instructive paracrine signals during organogenesis and thus take part in reciprocal cell-cell signaling required for the differentiation of other—often epithelial—cell types, for example during epidermal, oral, intestinal and kidney development^29–34^. Finally, fibroblasts are known to hold positional memory in the body, notably by expression of specific sets of Hox transcription factors in various sectors along the craniocaudal axis of the vertebrate embryo^35,36^.

To better understand the various roles of fibroblasts, it is important to gain insights into how heterogenous they are, as well as the functional consequences emanating from fibroblast heterogeneity. At least in part, research into fibroblast heterogeneity has however been hampered by ambiguous and diversified cell classification with regard to, for example, lineage, morphology, location or growth characteristics. An important step towards a deeper and more detailed characterization of fibroblasts can however now be taken thanks to single-cell transcriptomics, which complements the detailed anatomical and morphological knowledge with insights into the molecular profiles of fibroblasts at unprecedented depth and scale.

## Evolution from morphological to molecular characterization of fibroblasts

In normal adult tissues, fibroblasts appear spindle- or stellate-shaped with an oval nucleus and a distinct endoplasmic reticulum (ER)^3,4^ (for review see ref. ^27^). While these features are helpful to localize the cells anatomically, they provide few clues to origin, molecular composition, heterogeneity and relationship to other cell types. Although not uniquely expressed on fibroblast, certain proteins, such as vimentin (VIM (protein), *Vim* (gene/mRNA mouse)) and fibroblast specific protein 1 (FSP1, *S100a4*), have served as useful markers to identify fibroblasts by immunohistochemical techniques^37,38^. With the advent of technologies that allow the gene expression patterns of individual cells to be resolved at substantial depth, i.e., single-cell RNA-sequencing (scRNA-seq) (see Box 1 for a detailed description of the technology and potential caveats and pitfalls), an additional important step could be taken in the quest to better characterize fibroblasts along with all other cell types that make up our organs. Hence, the traditional morphological classification of cell types is now being complemented by molecular classifications, foremost based on gene expression.

Following from the first cDNA microarray-based study of fibroblast heterogeneity^24^, transcriptional analysis of fibroblast was conducted using bulk mRNA isolates obtained from multiple cells^39–41^. This work provided molecular information at the organ level and initial insights into inter-organ transcriptional heterogeneity of fibroblasts. From these and other studies, certain molecular markers were proposed to distinguish fibroblasts from other cell types, including VIM, platelet derived growth factor receptor-alpha (PDGFRA, *Pdgfra*), fibroblast activation protein-alpha (FAP, *Fap*), FSP1; and CD90 (*Thy1*). Subsequently, scRNA-seq datasets have been obtained from fibroblasts from essentially all major organs in the mouse and human (see Supplementary data 1 for a compilation of select scRNA-seq studies covering fibroblasts). When fibroblasts were collected and sequenced as part of broader atlas projects, they were occasionally called by other names, such as *stromal cells*, *mesenchymal stem cells, myofibroblasts*^42^ and *unknown mesenchymal* cells^43^, illustrating the prevailing ambiguities regarding markers and cell nomenclature. Some studies have been devoted more specifically to fibroblasts and their involvement in cancer, fibrotic diseases^44,45^ or development^46^. We have ourselves compared fibroblasts across healthy adult organs in the mouse^47^, whereas others have reported multi-organ comparisons between mice and humans^48^.

What have we learned from these scRNA-seq studies thus far? As a brief account (more specific examples and discussion are provided below), one lesson is that no single marker appears capable to discriminate all fibroblasts from all other cell types across organs; combinations of markers are needed. A second lesson is that organotypic fibroblast heterogeneity is profound^47^, although certain marker combinations may point to fibroblast subtypes that cross organ boundaries. Examples include peptidase inhibitor 16 (*Pi16*) and collagen type 15 alpha-1 (*Col15a1*) expression, which define subtypes present in several organs^48^, a *Wif1*^+^*Comp1*^+^ fibroblast subtype observed in heart valve as well as skeletal muscle perimysial fibroblasts, and two fibroblast subtypes present in both colon and bladder, defined by *Tnc*^+^*Cd34*^−^ and *Tnc*^-^*Cd34*^+^ expression, respectively^47^. Thirdly, insights into the specialized physiological functions of various fibroblast subtypes are emerging, for example in human skin, where some fibroblast subtypes appear specialized for ECM production and others for immunological and antimicrobial activities^49^, or in mouse skin, where fibroblast subtypes have been identified that participate in fibroblast growth factor (FGF) and Wnt signaling with hair follicle epithelial stem cells during cycling of the hair follicle^50^. In the mouse intestine, specific intestinal fibroblast subtypes are involved in Wnt signaling that regulates intestinal epithelial stem cell differentiation^32,47,51,52^. Fourthly, insights into the specialized pathological functions of various fibroblast subtypes are emerging. For example, a rare intestinal fibroblast subtype that converts arachidonic acid into prostaglandin E2 appears to play a role in tumorigenesis in the mouse^33^. Fibroblasts in skeletal muscle express specific cytokines and proinflammatory factors, likely aiding in regeneration of myofibers following injury^53^. A fifth lesson relates to species similarities and differences, including how similar human and mouse fibroblasts are, which is of relevance for human translation of mouse data. While the number of studies comparing mouse and human fibroblasts “side-by-side” are still few^48,54–58^, analogous fibroblast subtypes and similar gene activation programs exist in mouse and human cancer-associated fibroblasts (CAFs)^48^. There also appears to be molecular similarities between perivascular brain fibroblasts in humans and mice^59–62^, as well as between human and mouse lung fibrosis-inducing CTHRC1^+^ fibroblast subtypes^63,64^. Notwithstanding these examples, more research will be required to precisely define the degree of fibroblast conservation between the humans and mice.

Given the current pace of acquisition of fibroblast scRNA-seq data, we will undoubtedly see rapid progress, resulting in more fine-grained views of fibroblast heterogeneity, as well as refinement of transcriptomic signatures for cell type demarcations, such as proposed pan-fibroblast signatures defined by *Pdgfra*, *Dpt* combined with *Pi16* or *Col15a1*^48^, or a 90-gene signature that demarcates fibroblasts from vascular mural cells^47^. Progress will also be aided by improvements on the bioinformatics side (see Box 1 regarding technological considerations for scRNA-seq studies). Going forward, it is important that novel fibroblast subtypes are rigorously characterized, not only bioinformatically through clustering algorithms, which can be somewhat arbitrary and should be seen as a framework for detailed analysis rather than final conclusions about cell types, but also anatomically using carefully annotated RNA and protein markers. Ideally, marker combinations that readily and reliably distinguish fibroblasts from other cell types in specific organs of interest should be identified and used.

Box 1 Single-cell RNA sequencing *Technology:*

Single-cell RNA sequencing (scRNA-seq) is a technology for genome-wide and quantitative analysis of the transcriptome of cells at single-cell resolution^146^. Different scRNA-seq techniques provide complementary advantages that can be exploited depending on the biological question^148^. Techniques allowing the parallel analysis of many thousands of cells (e.g., Drop-seq) are useful for the identification of the cellular diversity within complex organs or whole individuals, whereas methods aimed at sorting of individual cells, for example by FACS, combined with chemistry for efficient mRNA capture and amplification (e.g., SmartSeq2 and 3^149^) allow deep transcriptional profiling of rare cells. Regardless of technology, scRNA-seq data has the ability to resolve cellular heterogeneity within an analyzed sample of cells, in marked contrast to traditional RNA sequencing (bulk RNA-seq), which provides averaged information in which any heterogeneity is masked. ScRNA-seq therefore offers an unprecedented opportunity to define and demarcate a cell class, such as fibroblasts, from other cell classes/types and to map heterogeneity in adult homeostasis, development and disease.

*Caveats and pitfalls:*

There are certain caveats with the current single-cell transcriptomic technologies. Loss of certain cell types during single-cell sample preparation represents a common problem; fragile cells do not cope well with the dissociation protocols and thus become under-represented or lost. Similarly, cells firmly embedded within the ECM, such as pericytes and mesangial cells, are hard to extract as intact individual cells, leading to under-representation or contamination by transcriptomes from other cell types. Using unbiased collection of “alive-labeled” cells may lead to that rare cell populations are out-competed by the most abundant cell types. The apparent lack of fibroblasts in some organ cell atlas studies may reflect these caveats^107^. Single-nuclei RNA sequencing (snRNA-seq) is an alternative single-cell transcriptomic strategy, which assesses in part immature mRNA molecules, thus circumventing some of the challenges with fragile or firmly embedded cells and can be applied to archived material^150^. SnRNA-seq is however prone to cross-contamination by “carry over” of mRNA from genes abundantly expressed by major cell types to the cell type of interest in the analyzed tissue. It should also be borne in mind that the algorithms for clustering of cells following scRNA-seq or snRNA-seq, such as Uniform Manifold Approximation and Projection (UMAP) and t-distributed stochastic neighbor embedding (tSNE), are machine learning-based and somewhat arbitrary, and the final clustering is dependent on the cellular composition of the sample at the starting point. This element of arbitrariness is likely to contribute to that different studies in some cases produce different subtype annotations, as they start from different cellular compositions of the starting samples. Comparison of transcriptomic datasets generated at different platforms (for example Drop-seq versus SmartSeq2/3) is also not yet straightforward, for example due to different sequencing depths, different mRNA/cDNA amplification rates, and the presence or absence of UMIs (unique molecular identifiers) and can generate erroneous conclusions.

*Considerations before initiating a scRNA-seq study:*

With the aim to identify a specific cell (sub)-type by scRNA-seq, it is important to consider whether it will be abundant enough to be captured by for example Drop-seq, or whether cell enrichment via cell surface marker expression (antibody-panning) or FACS sorting of fluorescence-labelled cells in transgenic models (see Box 2) will be required. Another consideration is the size of the cells, if FACS sorting is used. Some cells, such as cardiomyocytes or mature adipocytes^151^, are large and therefore lost or underrepresented following FACS sorting, and as discussed above some cell types are also generally more fragile. A third consideration is the choice of cell dissociation protocol, which needs to be “powerful” enough to yield single cells but should also be “minimal” enough not to impair cell and thus mRNA quality. Such protocols generally need to be fine-tuned for each organ and cell type. Fourth, in the subsequent bioinformatic analysis, attention needs to be paid not only to cell doublets, but also to cross-contamination (carry over) from other cell types (see above) as well as a concise appreciation of the lower limit of reliable gene-count signal, i.e., where does the noise level start. Finally, there may be situations where large-scale scRNA-seq experiments are not feasible for financial or other reasons, and if well-curated transcriptomic annotations have been made for cell types in the organ under study, bulk transcriptomic analysis may be a more affordable option, and observed expression changes can be related back to the cell type(s) of interest using prior knowledge about transcriptomes in the particular cell type(s). An informative example of this strategy was provided in a recent analysis of brain fibroblasts in amyotrophic lateral sclerosis^61^.

## Fibroblast heterogeneity within and between organs

As mentioned above, scRNA-seq data now allow fibroblast heterogeneity and relationship with as well as demarcation to other cell types to be decoded with unprecedented speed and precision. When discussing progress in these areas, we were inspired by the “*The Ancestor’s Tale*” by Richard Dawkins and Yan Wong, in which the authors describe our evolutionary history from the perspective of a human first discussing with its closest relatives, i.e., the extinct hominins, and then proceeding gradually to evolutionarily more distant relatives, ending with *Archaea*. In the same vein, we first discuss fibroblast heterogeneity within and between organs and then proceed to discuss relationships of fibroblasts to other connective tissue and vasculature cell types with whom fibroblasts share functional similarities and possible molecular relationships.

### Intra-organ fibroblast heterogeneity

**Fig. 1:**
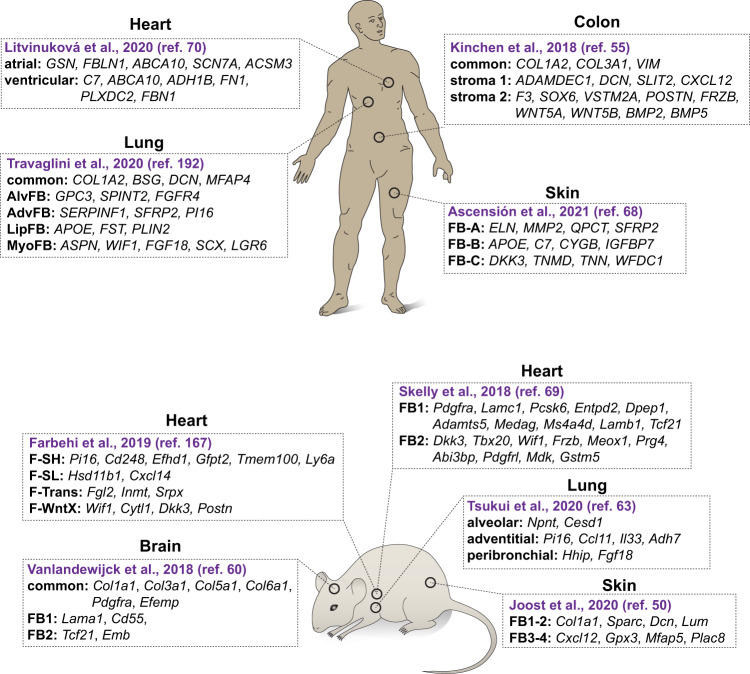
Schematic depiction of examples of intra-organ fibroblast heterogeneity in humans and mice based on scRNA-seq analysis. Examples of gene enrichment for specific fibroblast subtypes that were defined in each study are shown. Ascensión et al.^68^ summarizes four individual studies. AlvFB alveolar fibroblasts, AdvFB adventitial fibroblasts, LipFB lipofibroblasts, MyoFB myofibroblasts (sub-class: fibromyocytes), F-SH Sca1-high fibroblasts, F-SL Sca1-low fibroblasts, F-Trans transitory fibroblasts, F-WntX Wnt expressing fibroblasts. The annotations of fibroblast subtypes as well as their marker genes were compiled from the respective original works. If marker genes were not explicitly stated in the original paper, they were interpreted and derived from the available data presented in the figures. No additional analysis of the raw data was performed.

Fibroblast heterogeneity spans species, organ and developmental stage boundaries. It has long been recognized that different fibroblast populations reside simultaneously in the same organ (for review see refs. ^65,66^). For example, several studies have advanced our understanding of fibroblast subtypes in the skin (Fig. 1). In mice, fibroblasts residing in the upper (papillar) layer express alpha-8 integrin (*Itga8*) and dipeptidyl peptidase 4 (CD26, *Dpp4*), whereas fibroblasts in the lower (reticular) layer express podoplanin (*Pdpn*) and delta-like non-canonical Notch ligand 1 (*Dlk1*)^67^. One study identified four fibroblast subtypes in the adult mouse skin (FIB1-4), which based on marker expression (*Dcn*, *Gpx3*, *Sparc*, and *Plac8*, respectively), were found to occupy three distinct anatomical localization (dermis, hypodermis and adventitia)^50^. Two of the subtypes (FIB1 and 2), likely representing different states of the same fibroblast subtype, respond transcriptionally to different cell cycle stages in the hair follicle^50^. In humans, one study suggests that skin fibroblasts segregate into six transcriptionally distinct cell clusters, of which one (expressing *DPP4*) is the main ECM-producing cell type^49^. In a meta-analysis of human skin fibroblast transcriptomes, 10 subtypes formed three major groups (A-C, marked by *MMP2*, *IGFBP7* and *SFRP1* expression, respectively)^68^, of which group A appears specialized for ECM production and group B in immune surveillance^68^. In the mouse heart, two major fibroblast subtypes, presumably derived from endo- and epicardium, were identified and shown to express *Lamb1* and *Dkk3*, respectively^69^. Heart fibroblasts also express *Csf1*, *Vegfa*, *Igf1* and *Fgf2*, indicating active paracrine signaling to other cell types in the heart^69^. The human adult heart was found to contain seven fibroblast subtypes, two of which show enrichment in atria and ventricles and can be distinguished by expression of *SCN7A* and *CFH*, respectively, and two (called FB4 and FB5) which appear specialized in responding to transforming growth factor-β signaling and in ECM remodeling, respectively^70^. In the mouse lung, fibroblast subtypes have been characterized, one of which (a PDGFRa^+^Axin2^+^ subtype) responds by differentiating into myofibroblasts following injury^71,72^. An analysis of the human lung revealed five fibroblast subtypes, one of which (*CTHRC1*^+^) promotes lung fibrosis in COVID-19 patients^64^. Interestingly, a similar *Cthrc1*^+^ fibroblast subtype drives lung fibrosis in the mouse^63^. Two fibroblast subtypes with different injury-response features were identified in the synovial tissue of joints in the mouse: an immune effector subtype expressing fibroblast activation protein-α (FAPα)^+^ as well as THY1^+^ and a tissue destructive FAPα^+^THY1^−^ subtype^73^. Depletion of the FAPα^+^ fibroblasts reduced bone erosion and inflammation in a mouse arthritis model^73^. Another study identified NOTCH3 activity as a driver of inflammation in THY1^+^ synovial fibroblasts^44^. Intra-organ fibroblast heterogeneity is not confined to mammals but has also been noted in zebrafish where different *pdgfra*-positive fibroblast subpopulations take part in lymphangiogenesis^46^, or in the axolotl where several fibroblast populations were found amongst blastema-cells during limb regeneration^74,75^.

In addition to the distinction between fibroblast entities by the presence/absence of specific singular markers, intra-organ fibroblast heterogeneity may also reflect cell zonation, which is defined as gradual cellular phenotypic transitions along an anatomical or functional axis. For example, zonation of hepatocytes and endothelial cells occurs along the porto-central axis in the liver lobule^76,77^ and endothelial and mural cell zonation is observed along the arterial-venous axis in brain vasculature^60^. There are anatomical and physiological reasons to believe that fibroblast zonation (in addition to the Hox transcription code, discussed above) would make sense, for example along the muscle-tendon axis or along the crypt-villus and crypt-surface axes of the small and large intestine^32,34^. By identifying genes with gradient or nested transcription profiles across adjacent tissue areas/volumes, fibroblast zonation will potentially be uncovered and shown to be instrumental for generation and maintenance of proper tissue architecture in multiple organs.

### Inter-organ fibroblast heterogeneity

**Fig. 2:**
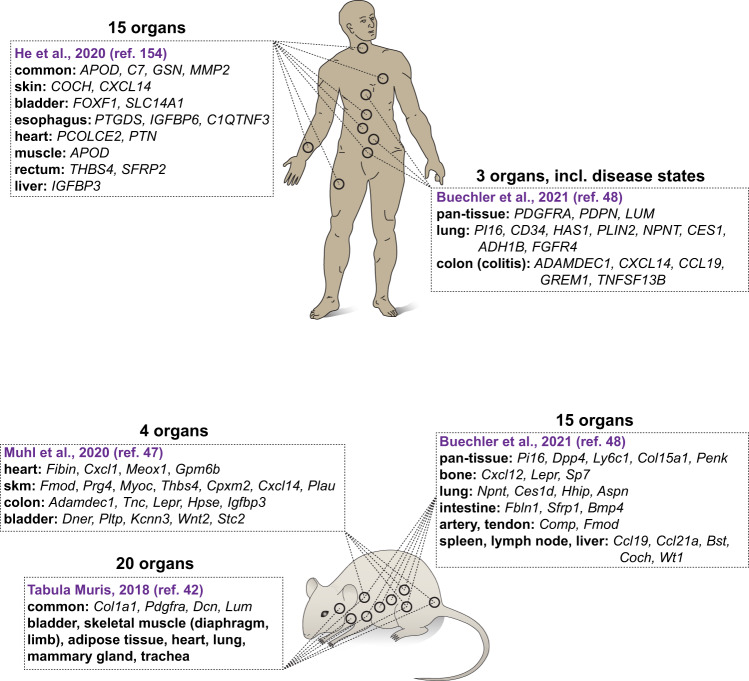
Schematic depiction of examples of inter-organ fibroblast heterogeneity in humans and mice, based on scRNA-seq analysis. Examples of gene enrichment for specific fibroblast subtypes that were defined in each study are shown. Studies that specifically investigate the inter-organ heterogeneity of fibroblasts were selected. a alveolar, p peribronchial, hi PDGFRα-high, rp red pulp. The annotations of fibroblast subtypes as well as their marker genes were compiled from the respective original works. If marker genes were not explicitly stated in the original paper, they were interpreted and derived from the available data presented in the figures. No additional analysis of the raw data was performed.

The first genome-wide transcriptional information on inter-organ fibroblast heterogeneity was provided through cDNA microarray analysis of cultures of human skin fibroblasts established from different ages and anatomical locations^24^. Interestingly, despite the concern that in vitro culturing may have influenced gene expression patterns, these fibroblasts retained a certain degree of transcriptional memory of their origin, including *HOX* gene expression patterns^24^. In a cross-comparison of mouse fibroblasts isolated directly from different muscular organs and analyzed by scRNA-seq, it was evident that differential expression of genes related to the ECM—the matrisome—is a major feature of inter-organ fibroblast transcriptomic heterogeneity^47^ (Fig. 2). This information is consistent with previous evidence that fibroblasts at different anatomical locations and at different ages tailor a unique composition of the ECM^24^. An important topic for further analysis is to understand the genetic and/or epigenetic underpinnings of how fibroblasts of different organs regulate their gene expression in order to achieve spatial/temporal-specific ECM profiles.

## The relationship between fibroblasts and other cell types in connective tissue and vasculature

### Specialized connective tissue cell types

Several connective tissues harbor highly specialized cell types: adipocytes in fat, chondrocytes in cartilage, and osteocytes/blasts in bone. There are both in vivo and in vitro data suggesting that these cell types are lineage-related and share a common mesenchymal and fibroblast-like origin, and it is therefore of interest to explore how related the differentiated cells are to each other and to fibroblasts at the molecular level. Adipocytes are designed to store, release, and burn lipids at different relative rates depending on their subtype (white, brown, and beige/brite). Several scRNA-seq studies of adipocytes have been conducted, although adult white adipocytes are challenging to handle due to their large size and fragility (for review see ref. ^78^). From available data, it is apparent that the transcriptomes of differentiated fibroblasts and adipocytes are readily distinguishable by the adipocyte transcripts that encode proteins involved in lipid handling. The resemblance between the adipocyte precursor cell (preadipocyte) and fibroblasts is however close, although relationships between preadipocytes and pericytes, discussed below, have also been proposed (see ref. ^79^ and references therein). Chondrocytes are morphologically highly similar to fibroblasts, but express several unique proteins, notably a number of ECM proteins contained within cartilage, including collagen type 2^80^ (for review see ref. ^81^). Beyond the highly specific markers of adipocytes, chondrocytes and osteoblasts, further analyses are needed to define transcriptional similarities and differences between these cells, their precursors, and fibroblasts.

### Vascular mural cells

Vasculature courses through and is sometimes even counted as a component of connective tissue. Blood vessels consist of an inner tubing of endothelial cells surrounded by mural cells. The latter are either vascular smooth muscle cells (VSMCs), located in larger vessels (arteries, arterioles and veins), or pericytes, which coat capillaries and some venules and are transcriptomically related to venous VMSCs^60^. VSMCs contain a contractile machinery involving several proteins, among them alpha-smooth muscle actin *(*ASMA*, Acta2)*, which is a highly expressed and often-used smooth muscle cell marker. Pericytes probably have multiple and organ-specific functions, for example in the brain where they are essential for the maturation and maintenance of the blood-brain barrier^82,83^. Molecularly, pericytes can be distinguished from VSMCs by for example abundant expression of potassium inwardly rectifying channel subfamily J member 8 (*Kcnj8*) and absent or very low (relative to VSMCs) expression of transcripts encoding the smooth muscle contractile machinery^60^. Fibroblasts reside on the outside of the mural coat in many vessel types. In the largest arteries and veins, fibroblasts form an anatomically discernable vascular layer, the adventitia. Pericytes have a morphology somewhat resembling that of fibroblasts, and it has been proposed that pericytes are in fact specialized perivascular fibroblasts^84^. Live imaging in zebrafish embryos indeed suggest that mural cells derive from perivascular fibroblast-like cells^85^. ScRNA-seq analysis of pericytes from several organs, including brain, lung, skeletal muscle, colon, bladder and heart has illustrated their relationship to fibroblasts^47,60^. From these studies, it seems clear that a distinct demarcation between fibroblast and pericyte transcriptomes can be made, including differences in ECM-related transcription profiles. For example, pericytes express specific ECM-binding proteins, such as basal cell adhesion molecule (BCAM, *Bcam*), but generally low levels of collagens in comparison to fibroblasts, with the exception of collagen type 4 (*Col4a1, Col4a2*), which are components of the microvascular basement membrane to which the pericytes contribute^47^. The molecular demarcation between VSMCs and fibroblasts also seems distinct, with several genes uniquely expressed in one or the other cell type. Regarding ECM production, VSMCs express low amounts of collagens, similar to pericytes, but high amounts of elastin (*Eln*), and certain other ECM proteins of presumed relevance for the resilience of the arterial wall^47^. Genes encoding proteins of the smooth muscle contractile machinery, such as *Acta2*, myosin heavy chain 11 (*Myh11*), transgelin (*Tagln)*, calponin 1 (*Cnn1*), leiomodin 1 (*Lmod1*) and regulator of calcineurin 2 (*Rcan2*), are highly expressed by VSMCs, distinguishing them from hitherto analyzed quiescent fibroblasts, although these genes may be re-expressed (albeit at lower levels than in VSMCs) when fibroblasts become activated into myofibroblasts during pathological conditions or in normal fibroblasts located in organs with high deformation capacity, such as the urinary bladder^47,57^.

### Cell types with features of both fibroblasts and pericytes

**Fig. 3:**
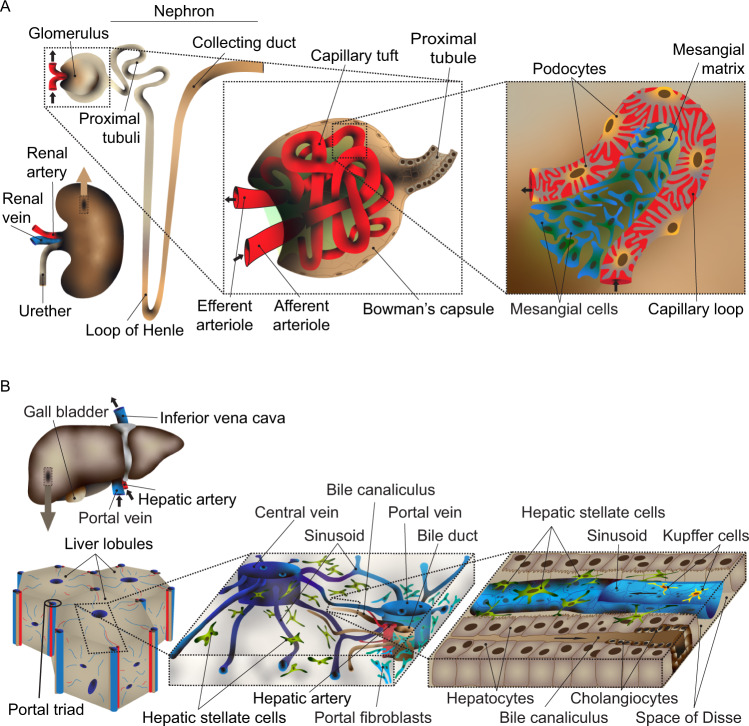
Cell types with features of both fibroblasts and pericytes. **A** Schematic depiction of the kidney with the nephron and glomerulus. In the glomerulus, the position of the mesangial cells close to a capillary loop is shown. **B** Schematic depiction of the liver with the lobules and portal triads. The position of the hepatic stellate cells along the sinusoid is shown.

While in most cases fibroblasts and pericytes can be readily distinguished by their distinct gene expression profiles, certain cell types appear to exhibit characteristics of both. In the kidney glomerulus (Fig. 3A), mesangial cells display intermediate features between those of pericytes and fibroblasts, VSMC and myofibroblasts^86^. Mesangial cells have indeed been proposed to be a specialized type of pericytes^87,88^, a notion supported by their lineage-dependence on PDGFB-PDGFRB signaling^89–91^, which is shared with other mural cell lineages. Other reports however claim that mesangial cells are distinct from pericytes^92^. Mesangial cells lack the typical cellular morphology and vascular basement membrane embedment of other capillary pericytes, and the fact that they deposit a rich ECM (called mesangial matrix) may be more in line with a fibroblastic than pericytic nature. However, no other pericyte-like cells are present within the glomerulus, and if a generic pericyte function were needed there, it would have to be provided by the mesangial cells. Currently, several kidney scRNA-seq studies have claimed to identify (and hence molecularly characterize) mesangial cells, but links between cellular heterogeneity to precise anatomical location were lacking^93–95^. However, one study succeeded in distinguishing between intra- and extraglomerular mesangial cells through their unique scRNA-seq profile and in situ analysis of relevant markers^58^. Notably, the intraglomerular mesangial cells expressed desmin (*Des*) and *Acta2*, i.e., markers of mural cells, but also *Pdgfra* and *Pi16*, which are fibroblast markers typically absent in mural cells^58^.

Hepatic stellate cells (HSCs) were originally discovered in the 1870s by Karl Wilhelm von Kupffer, who called them Sternzellen (star cells)^96^ and portrayed them as a specialized type of pericyte by virtue of their anatomical position along the sinusoidal capillaries and in the perisinusoidal space (the space of Disse) between the endothelial cells and hepatocytes^97–100^ (Fig. 3B). Like pericytes in other organs, the HSCs contact capillary endothelial cells, but unlike both typical pericytes and glomerular mesangial cells, HSCs do not depend on PDGFB-PDGFRB signaling for their embryonic development^101^, possibly suggesting a non-mural identity. HSCs were recently characterized by scRNA-seq and found to be heterogeneous across the healthy liver lobule, with distinct molecular markers expressed at the periportal and central locations, respectively^102^. Similar to mesangial cells, HSCs appear to express markers of both mural cells (*Des*) and fibroblasts (*Pdgfra*), suggesting an identity intermediate between these two cell types.

### Annotation of ambiguous cell types

The emerging transcriptomic profiles of fibroblasts and the other cell types discussed above can be useful to shed light on the nature of recently identified cell types, for which annotation has been problematic, or remains controversial. A novel type of pericyte involved in spinal cord repair has been reported^103,104^. While these cells share some markers with brain pericytes and locate in the proximity of blood vessels, other markers may argue that these cells instead are perivascular fibroblasts^105^, a notion receiving support from scRNA-seq data^60^. Lineage-tracing studies likewise suggest a fibroblast origin of cells forming spinal cord fibrotic scars^106^, but a final conclusion will have to await a more extensive analysis of all cell types present in the spinal cord vasculature. Kidney fibroblasts were recently annotated in a kidney scRNA-seq study based on the expression of a singular marker, FSP1 (*S100a4*)^107^. However, cross-organ comparison provides little support for these cells being fibroblasts, but there is instead ample evidence for similarity with macrophages^47,108^, which, along with smooth muscle cells, also express FSP1. Another study instead annotated kidney fibroblasts in both human and mouse as positive for PDGFRA, PDGFRB, maternally expressed 3 (MEG3), Scavenger Receptor Class A Member 5 (SCARA5) and numerous collagens^109^, which are markers found also in fibroblasts from several other organs^47^. Further studies are thus needed to establish the identity and possible heterogeneity of kidney fibroblasts. Some of the annotations in the large-scale analysis presented in Tabula Muris^42^ may also need to be revised, notably some cell populations defined as myofibroblasts should rather be referred to as mural cells (pericytes and/or VSMCs). A novel lung cell type characterized by high PDGFRB expression and expansion in response to experimental lung injury was recently identified^110^. The authors posed the question whether these cells were pericytes, a notion supported by the extensive transcriptomic similarity to cells independently annotated as lung pericytes^60^. These examples illustrate how the improved understanding of fibroblasts, pericytes and other vascular or connective tissue cell types may assist in fine-tuning annotations of cell types from various datasets.

## Lineage relationships between fibroblasts and other cell types

**Fig. 4:**
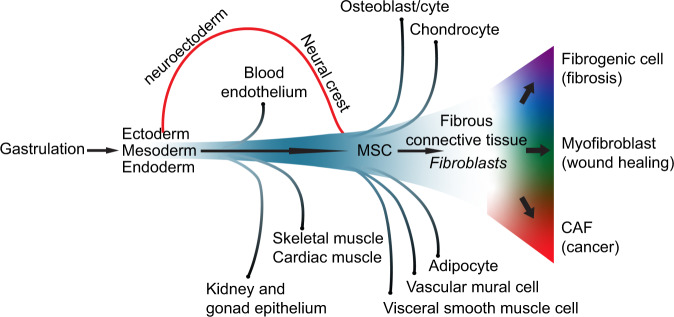
Schematic representation of the fibroblast lineage, with the origin in mesoderm and from ectoderm (via the neural crest) for fibroblasts in the head. Other cell types originating from the mesoderm are also depicted. MSC are shown as a transitory cell type that yield fibroblasts. In fibrosis, wound healing and cancer, fibroblasts likely progress further to become fibrogenic cells, myofibroblasts and CAFs, respectively.

The recent advances in transcriptomics can be productively interfaced with insights from lineage-tracing analysis to better understand fibroblast lineage relationships. Connective tissue is largely derived from mesoderm that via epithelial-to-mesenchymal transition (EMT) of the primitive epithelium generates embryonic mesenchyme, which in turns forms most connective tissues in the adult organism, including fibroblasts^65,111^ (Fig. 4). The vascular mural cells of most trunk tissues likewise have a mesodermal origin, arguing that a common progenitor for fibroblasts and vascular mural cells can be found somewhere in the mesodermal lineage^65^, a view supported by lineage-tracing analysis^112^. Fibroblasts and mural cells in the head are however in part derived from a different origin, the neuroectoderm (neural crest)^84^, and it will be interesting to learn to what extent transcriptomic differences between fibroblasts and mural cells in the head versus organs in the trunk reflect these different cellular origins. To establish fibroblast transcriptomes from different developmental stages will also be important to understand how different fibroblast subtypes dynamically change during development.

In a discussion on connective tissue lineage and possible stem/progenitor populations, it is difficult to avoid touching upon mesenchymal stem cells (MCS; a.k.a. mesenchymal stromal cells). MSCs were first mentioned in the 1960s in conjunction with bone marrow transplantation and explant studies^113,114^, and cells with the potential of differentiating into multiple connective tissue phenotypes, including adipose, cartilage and bone, were subsequently established in vitro from multiple tissue sources (for review see ref. ^115^). There has however been a lack of rigorous criteria to define MSCs^116^ and although efforts are made to define a set of commonly agreed markers^115,117^, there is still a somewhat bewildering literature regarding MSC cell lineage and differentiation capabilities. It should also be considered that most MSC differentiation experiments have been conducted in vitro, which makes it difficult to yet establish their true existence in vivo, both regarding abundance and locations, and to what extent they contribute to repair and remodeling of connective tissue, including fibroblasts. Several studies propose that pericytes may be in vivo equivalents to MSCs based on that MSCs can be established from explanted vascular fragments and by using a limited set of markers^118–120^. A strong similarity between MCSs and fibroblasts has however also been proposed^121^. It is increasingly clear that fibroblasts coat also small caliber vessels without a discernable adventitia and that fibroblasts and mural cells share many markers including the ones used for MSCs^47,60^. In sum, MCSs have been extracted from various tissues, but their true in vivo origin, i.e., whether they exist as a bona fide resident stem/progenitor cell in the tissue or derive through dedifferentiation of a tissue-resident fibroblast, pericyte or other cells, remains to be worked out.

Lineage-tracing in the mouse has shed new light on relationships between fibroblast subtypes and between fibroblasts and other cell types, although there are still some technical caveats and pitfalls with the technology, as discussed in Box 2. In skin, progenitors for both papillar and reticular fibroblasts, as well as for the papillar and reticular lineages individually, have been identified by the use of *Pdgfra*-CRE, *Blimp1*-CRE and *Dlk1*-CRE drivers, respectively^67^ (for review see ref. ^122^). Skin fibrosis has been lineage traced to an *En1*^+^ fibroblast population^123^. During wound healing, cells descending from a *Hic1*^+^ mesenchymal progenitor population generate extrafollicular fibroblasts^124^, and *Hic1* marks mesenchymal progenitors with functions in regeneration also in skeletal muscle^53^ and heart^125^. Interestingly, these cells express a number of pericyte markers (e.g., *Rgs5, Mcam, Notch3* and *Kcnj8*), possibly suggesting a lineage relationship between pericytes and regenerating fibroblasts^53^. In heart, mesenchymal progenitors yield *Pdgfra*^+^*Sca1*^−^ fibroblasts upon cardiac injury, and the transition towards the fibroblast state is regulated by *Hic1*^125^. Lineage-tracing of a *Pdgfra*^+^*Ly6a*^+^(Sca1) mesenchymal stromal progenitor cell in skeletal muscle indicates that it can give rise to both fibroblasts and adipocytes^126,127^ and be diverted towards an osteogenic fate by bone morphogenetic protein stimulation^128^. This may suggest a common mesenchymal progenitor for fibroblasts, adipocytes and osteoblasts.

Box 2 Reporter gene-based lineage-tracing and cell sorting strategies using transgenic mice *Technology:*

Lineage tracing and sorting of cells from transgenic mice can be achieved by expression of a reporter gene (fluorescent reporter genes such as eGFP and tdTomato or lacZ (beta-galactosidase)) in a cell type of interest. A reporter gene can be introduced into the mouse genome in two principal ways (Figure A). The reporter gene can: (1) be linked to a specific promoter element which should drive expression in a specific cell type and the combined promoter-gene construct randomly inserted in a transgenic mouse; or (2) be inserted into a specific genomic site, where its expression is driven from a nearby regulatory (promoter/enhancer) element. A third way to express a reporter gene in cells of interest is to insert it into the genome near a constitutive regulatory element (such as the Rosa26 locus) but preceded by a stop cassette which prohibits expression (Figure B). The stop cassette is flanked by recognition sequences (loxP sites) for CRE recombinase (or other excising enzymes such as FLP recombinase), and when the mouse carrying the silenced reporter gene is crossed with a mouse expressing CRE recombinase in the cell type of interest (introduced into the mouse genome by the same options as described above), reporter gene expression is activated in this cell type (Figure B). This CRE recombinase-based strategy is frequently used for lineage-tracing, as the reporter gene becomes permanently activated after the removal of the stop cassette, and all cells descending from an original reporter gene-expressing cell will express the reporter gene (Figure B).

*Caveats and pitfalls:*

There are however caveats for all reporter-based systems. If the promoter element does not perfectly recapitulate the expression of the endogenous gene, this leads to a reporter gene expression pattern not fully mimicking the endogenous expression pattern. For example, the endothelial specificity of VE-cadherin (*Cdh5*)-driven CRE recombinase expression might not be as high as anticipated, since scRNA-seq data show that perivascular fibroblasts in the brain also express *Cdh5* mRNA^60^. Likewise, the use of FSP1 (*S100a4*) to mark fibroblasts may be compromised by that it is also expressed by certain macrophages^152^. When cells are sorted for scRNA-seq experiments, this can lead to inclusion of cells where aberrant reporter gene expression occurs, or exclusion of cells which do express the endogenous gene but where the promoter element was not functional. Aberrant promoter activity is also a problem for CRE-based lineage-tracing studies; too extensive or too narrow expression of the CRE recombinase in relation to the cell type intended to be labelled leads to an incorrect starting point for a lineage-tracing analysis, which can result in too many or too few labelled cells in the original CRE-activated cell population (Figure C) (for examples, see Supplementary Data 1 and refs. ^153–225^).

## Fibroblasts and pathological conditions

Fibroblasts have been strongly implicated as the major fibrogenic cell type in pathological tissue fibrosis. 50 years ago, it was demonstrated that fibroblasts convert to myofibroblasts expressing certain smooth muscle cell markers, notably *Acta2*, when activated by tissue injury or stress^25^. While this transition is part of a normal wound-healing process that resolves over time, it can also result in persistent fibrosis, a severe medical condition with impaired function of organs, including the kidney, lung, liver and heart. A hallmark of fibrosis is the accumulation of myofibroblasts and excessive ECM deposition resulting in abnormal fibrous connective tissue (for review see refs. ^27,129^). While many studies point to fibroblasts as the source of the myofibroblast, the situation may not be that simple. Alternative suggested origins of myofibroblasts include endothelial cells, macrophages, mesothelial cells (the squamous epithelial lining of body cavities and their organs), pericytes, HSCs and MCSs. Summarizing the extensive body of original papers regarding the potential diverse origins of myofibroblasts goes beyond the scope of this review, and we refer interested readers to other literature on the topic^66,129,130^. Lineage-tracing has identified a *Gli1*^+^ mesenchymal progenitor population, which generates myofibroblasts and drives fibrosis in a sonic hedgehog (*Shh*)-dependent manner in kidney, lung, liver, heart and bone marrow^131–133^. The localization of the *Gli1*^+^ mesenchymal cells close to endothelial cells in the perivascular niche and their expression of *Cspg4* (NG2) may suggest a relationship to pericytes^131^. The partially conflicting propositions for the various cellular origins of myofibroblasts rest on lineage-tracing experiments in mice and may to some extent reflect methodological pitfalls, including leakiness in inducible CRE/loxP systems^134^ or aberrant expression of CRE recombinase (Box 2). It however remains a possibility that there is a multi-cell type (including non-fibroblastic) origin generating functionally distinct subpopulations of myofibroblasts. Heterogeneity of fibroblast origin and differentiation during tissue fibrosis is indeed supported by data from heart, lung and kidney^109,135,136^. Further careful assessment of transcriptomic data from resting fibroblasts and other normal cell types, comparisons with myofibroblast transcriptomes, and the analysis of transcriptional profile changes during myofibroblast generation^109^ should bring clarity to both origin and heterogeneity amongst pathological myofibroblasts.

In cancer research, it is increasingly realized that it is not only the properties of the genetically abnormal tumor cells that dictates the cancer’s severity, growth potential and ability to metastasize; the outcome is also influenced by the genetically normal stroma cells that populate all solid tumors at variable abundance. Among the latter, CAFs constitute an important cell population (for a recent consensus statement paper on CAFs, see ref. ^137^). Together with their produced matrix, CAFs often make up a significant volume of carcinomas, and they influence tumor growth and metastasis^27,28^. Similar to the situation with myofibroblasts and fibrosis, our knowledge about the origin and molecular characteristics of CAFs is still limited—they are in part “negatively” characterized by not expressing markers for other well-annotated cell types and not carrying the chromosomal abnormalities observed in the neoplastic tumor cells. To gain insights into the origin of CAFs is complicated, particularly in humans, and relies on inference from studies of earlier stages of cancer (hyperplasia and adenomas), and based on such studies, normal tissue fibroblasts have been implicated as a founding cell type for CAFs (for review see ref. ^138^). Lineage-tracing experiments in mice have resulted in different views on the cell type of origin for CAFs^137^, but the conclusions may suffer from problems with CRE expression specificity (Box 2), as discussed above for myofibroblasts. Cell transplantation experiments provide an additional source of information regarding the cellular origin of CAFs. In such experiments, asking whether a particular transplanted cell type ends up as CAFs in the tumor stroma, both MSCs and adipocytes appear to be able to generate CAFs in xenograft mouse tumor models^139–143^. As for myofibroblasts, it is an emerging view that distinct subpopulations of CAFs can be identified in a particular tumor stroma^45^, possibly indicating a multi-cell type origin of CAFs.

## Conclusions and future outlooks

At present, our understanding of fibroblasts is rapidly improving, and large-scale transcriptomic analyses at single-cell resolution allow us to better define fibroblasts, their heterogeneity and relationship to other types and classes of cells. While this undoubtedly is transforming our current understanding, it is important to also consider that there still is room for improvement regarding the current transcriptomic and transgenic technologies (see Box 1 and Box 2 for examples). Transcriptomic technologies indeed rapidly improve and become more affordable, which will lead to an acceleration in the accumulation of gene expression information with increasing granularity. Similarly, novel lineage-tracing methodologies are at the horizon, which rely less on promoter specificity but more on clonal evolution based on naturally occurring somatic mutations in cells^144,145^ or the introduction of traceable genetic markers^146^. Improved bioinformatic algorithms to identify cell state transitions and advances in spatial transcriptomics will likewise help deciphering lineage relationships and the exact anatomic location of fibroblast subsets^147^. Such studies may shed new light on lineage relationship and provide insights into whether differentiation yields only divergent fibroblast subsets from a founding cell population or whether there may also be convergence from different lineages towards a similar molecular phenotype, for example when fibroblasts of different origins experience similar physiological conditions and challenges.

Undoubtedly, gene expression signatures that define and demarcate fibroblasts will be refined and reveal further intra- and inter-organ fibroblast heterogeneity, advancing our functional understanding of fibroblasts and its related cell types. While this progress in principle is applaudable, it should be remembered that transcriptomic information, even when available with perfect fidelity for single cells, is not the sole basis of cellular heterogeneity. Cellular protein levels may deviate from the corresponding mRNA levels, and posttranslational protein modifications provide an additional level of molecular complexity and heterogeneity. Additionally, lipids, sugars and low molecular weight metabolites and solutes are likely to contribute to cellular heterogeneity independent of transcriptional control. Whereas single-cell analysis of other molecules than DNA and RNA currently has limited depth, we may expect technological advances also in these areas, and therefore our view and understanding of cellular (including fibroblast) heterogeneity will continue to evolve. Secondly, while the wealth of single-cell transcriptomic data can inspire efforts to define an expanding number of molecularly distinct fibroblast subpopulations, it must all the time be asked to what extent the molecular differences are physiologically meaningful; some of the identified cell “subtypes” may reflect states on a developmental trajectory or responses to stress or injury rather than stable functional specializations at steady state. To tease out specific physiological functions for steady state differences in fibroblasts will undoubtedly take longer, but it remains an important future research avenue and is increasingly feasible. It is possible that the fibroblast cell class in due time may be subdivided into functionally meaningful fibroblast subtypes, some of which execute organ-specific physiological functions, while other subtypes may transcend organ boundaries and carry out functions that are not organ-specific, such as the involvement in immune responses or wound healing. The latter may lead to a terminology similar to the one established for T-cells, for which we now recognize a set of functionally distinct subtypes, including T-regs, CD8^+^ T-cells, CD4^+^ T-cells and double-negative T-cells. It will be interesting to see how these endeavors will pan out, but it is certainly likely that the fibroblast will be a well-defined and annotated class of cells for which heterogeneities across and within organs as well as developmental and pathological states are understood in a foreseeable future.

## Supplementary information

Description of additional supplementary files

Supplementary Dataset 1

## References

[1] Virchow, R. *Die Cellularpathologie in Ihrer Begruendung auf Physiologische und Pathologische Gewebelehre* (Hirschwald A, Berlin, Germany, 1858).

[2] Bloom, W. & Fawcett, DW. *A Textbook of Histology*. (CRC Press, 1998).

[3] Duvall, M. *Atlas d’Embryologiee*. (ed. Masson, G) (Libraire de l’académie de médecine, Paris, France, 1879).

[4] Movat HZ, Fernando NVP (1962). The Fine Structure of Connective Tissue I. The Fibroblast. Exp. Mol. Pathol..

[5] Ziegler, E. *General Pathology*. (William Wood and Company, 1896).

[6] Maximow A (1905). Über die Zellformen des lockeren Bindegewebes. Arch. f.ür. mikroskopische Anat..

[7] Lewis, M. *Development of connective-tissue fibers in tissue cultures of chick embryos* (Carnegie Institution of Washington, Washington, 1917).

[8] Stearns ML (1940). Studies on the development of connective tissue in transparent chambers in the rabbit’s ear. I. Am. J. Anat..

[9] Stearns ML (1940). Studies on the development of connective tissue in transparent chambers in the rabbit’s ear. II. Am. J. Anat..

[10] Porter KR (1964). Cell fine structure and biosynthesis of intracellular macromolecules. Biophysical J..

[11] Chesney J, Bucala R (1997). Peripheral blood fibrocytes: novel fibroblast-like cells that present antigen and mediate tissue repair. Biochemical Soc. Trans..

[12] Bucala R, Spiegel LA, Chesney J, Hogan M, Cerami A (1994). Circulating fibrocytes define a new leukocyte subpopulation that mediates tissue repair. Mol. Med..

[13] Chong SG, Sato S, Kolb M, Gauldie J (2019). Fibrocytes and fibroblasts—Where are we now. Int. J. Biochem. Cell Biol..

[14] Abercrombie M, Heaysman JEM (1953). Observations on the social behaviour of cells in tissue culture. I. Speed of movement of chick heart fibroblasts in relation to their mutual contacts. Exp. Cell Res..

[15] Hayflick L (1965). The limited in vitro lifetime of human diploid cell strains. Exp. Cell Res..

[16] Shay JW, Wright WE (2000). Hayflick, his limit, and cellular ageing. Nat. Rev. Mol. Cell Biol..

[17] Shih C, Padhy LC, Murray M, Weinberg RA (1981). Transforming genes of carcinomas and neuroblastomas introduced into mouse fibroblasts. Nature.

[18] Takahashi K, Yamanaka S (2006). Induction of pluripotent stem cells from mouse embryonic and adult fibroblast cultures by defined factors. Cell.

[19] Rheinwald JG, Green H (1975). Serial cultivation of strains of human epidermal keratinocytes: the formation keratinizing colonies from single cells. Cell.

[20] Evans MJ, Kaufman MH (1981). Establishment in culture of pluripotential cells from mouse embryos. Nature.

[21] Karsdal, M. *Biochemistry of collagens, laminins and elastin*. (Academic Press, 2019). 10.1016/C2018-0-00074-2.

[22] Hynes RO, Naba A (2012). Overview of the matrisome-An inventory of extracellular matrix constituents and functions. Cold Spring Harb. Perspect. Biol..

[23] Schor SL, Schor AM (1987). Clonal heterogeneity in fibroblast phenotype: Implications for the control of epithelial-mesenchymal interactions. BioEssays.

[24] Chang HY (2002). Diversity, topographic differentiation, and positional memory in human fibroblasts. Proc. Natl Acad. Sci. USA.

[25] Gabbiani G, Ryan GB, Majne G (1971). Presence of modified fibroblasts in granulation tissue and their possible role in wound contraction. Experientia.

[26] Pakshir P (2020). The myofibroblast at a glance. J. Cell Sci..

[27] Kalluri R (2016). The biology and function of fibroblasts in cancer. Nat. Rev. Cancer.

[28] Pietras K, Östman A (2010). Hallmarks of cancer: Interactions with the tumor stroma. Exp. Cell Res..

[29] el Ghalbzouri A, Lamme E, Ponec M (2002). Crucial role of fibroblasts in regulating epidermal morphogenesis. Cell Tissue Res..

[30] Costea DE, Johannessen AC, Vintermyr OK (2005). Fibroblast control on epithelial differentiation is gradually lost during in vitro tumor progression. Differentiation.

[31] Liu Y (2011). Cellular and molecular mechanisms of renal fibrosis. Nat. Rev. Nephrol..

[32] Degirmenci B, Valenta T, Dimitrieva S, Hausmann G, Basler K (2018). GLI1-expressing mesenchymal cells form the essential Wnt-secreting niche for colon stem cells. Nature.

[33] Roulis M (2020). Paracrine orchestration of intestinal tumorigenesis by a mesenchymal niche. Nature.

[34] Shoshkes-Carmel M (2018). Subepithelial telocytes are an important source of Wnts that supports intestinal crypts. Nature.

[35] Alexander T, Nolte C, Krumlauf R (2009). Hox Genes and Segmentation of the Hindbrain and Axial Skeleton. Annu. Rev. Cell Dev. Biol..

[36] Rinn JL, Bondre C, Gladstone HB, Brown PO, Chang HY (2006). Anatomic demarcation by positional variation in fibroblast gene expression programs. PLoS Genet..

[37] Dulbecco R, Allen R, Okada S, Bowman M (1983). Functional changes of intermediate filaments in fibroblastic cells revealed by a monclonal antibody. Proc. Natl Acad. Sci. USA.

[38] Strutz F (1995). Identification and characterization of a fibroblast marker: FSP1. J. Cell Biol..

[39] Heruth DP, Gibson M, Grigoryev DN, Zhang LQ, Ye SQ (2012). RNA-seq analysis of synovial fibroblasts brings new insights into rheumatoid arthritis. Cell Biosci..

[40] Nota B (2014). RNA sequencing of creatine transporter (SLC6A8) deficient fibroblasts reveals impairment of the extracellular matrix. Hum. Mutat..

[41] Lahiry P (2011). Transcriptional profiling of endocrine cerebro-osteodysplasia using microarray and next-generation sequencing. PLoS ONE.

[42] Schaum N (2018). Single-cell transcriptomics of 20 mouse organs creates a Tabula Muris. Nature.

[43] Green CD (2018). A Comprehensive Roadmap of Murine Spermatogenesis Defined by Single-Cell RNA-Seq. Developmental Cell.

[44] Wei K (2020). Notch signalling drives synovial fibroblast identity and arthritis pathology. Nature.

[45] Bartoschek M (2018). Spatially and functionally distinct subclasses of breast cancer-associated fibroblasts revealed by single cell RNA sequencing. Nat. Commun..

[46] Wang G (2020). Specific fibroblast subpopulations and neuronal structures provide local sources of Vegfc-processing components during zebrafish lymphangiogenesis. Nat. Commun..

[47] Muhl L (2020). Single-cell analysis uncovers fibroblast heterogeneity and criteria for fibroblast and mural cell identification and discrimination. Nat. Commun..

[48] Buechler MB (2021). Cross-tissue organization of the fibroblast lineage. Nature.

[49] Vorstandlechner V (2020). Deciphering the functional heterogeneity of skin fibroblasts using single-cell RNA sequencing. FASEB J..

[50] Joost S (2020). The Molecular Anatomy of Mouse Skin during Hair Growth and Rest. Cell Stem Cell.

[51] Stzepourginski I (2017). CD34+ mesenchymal cells are a major component of the intestinal stem cells niche at homeostasis and after injury. Proc. Natl Acad. Sci. USA.

[52] Valenta T (2016). Wnt Ligands Secreted by Subepithelial Mesenchymal Cells Are Essential for the Survival of Intestinal Stem Cells and Gut Homeostasis. Cell Rep..

[53] Scott RW, Arostegui M, Schweitzer R, Rossi FMV, Underhill TM (2019). Hic1 Defines Quiescent Mesenchymal Progenitor Subpopulations with Distinct Functions and Fates in Skeletal Muscle Regeneration. Cell Stem Cell.

[54] Elyada E (2019). Cross-species single-cell analysis of pancreatic ductal adenocarcinoma reveals antigen-presenting cancer-associated fibroblasts. Cancer Discov..

[55] Kinchen J (2018). Structural Remodeling of the Human Colonic Mesenchyme in Inflammatory Bowel Disease. Cell.

[56] Merrick D (2019). Identification of a mesenchymal progenitor cell hierarchy in adipose tissue. Science.

[57] Yu Z (2019). Single-cell transcriptomic map of the human and mouse bladders. J. Am. Soc. Nephrol..

[58] He B (2021). Single-cell RNA sequencing reveals the mesangial identity and species diversity of glomerular cell transcriptomes. Nat. Commun..

[59] Zeisel A (2018). Molecular Architecture of the Mouse Nervous System. Cell.

[60] Vanlandewijck M (2018). A molecular atlas of cell types and zonation in the brain vasculature. Nature.

[61] Månberg A (2021). Altered perivascular fibroblast activity precedes ALS disease onset. Nat. Med..

[62] Liu J (2021). A human cell type similar to murine central nervous system perivascular fibroblasts. Exp. Cell Res..

[63] Tsukui T (2020). Collagen-producing lung cell atlas identifies multiple subsets with distinct localization and relevance to fibrosis. Nat. Commun..

[64] Melms JC (2021). A molecular single-cell lung atlas of lethal COVID-19. Nature.

[65] LeBleu VS, Neilson EG (2020). Origin and functional heterogeneity of fibroblasts. FASEB J..

[66] Plikus MV (2021). Fibroblasts: Origins, definitions, and functions in health and disease. Cell.

[67] Driskell RR (2013). Distinct fibroblast lineages determine dermal architecture in skin development and repair. Nature.

[68] Ascensión AM, Fuertes-Álvarez S, Ibañez-Solé O, Izeta A, Araúzo-Bravo MJ (2021). Human Dermal Fibroblast Subpopulations Are Conserved across Single-Cell RNA Sequencing Studies. J. Investig. Dermatol..

[69] Skelly DA (2018). Single-Cell Transcriptional Profiling Reveals Cellular Diversity and Intercommunication in the Mouse Heart. Cell Rep..

[70] Litviňuková M (2020). Cells of the adult human heart. Nature.

[71] Rock JR (2011). Multiple stromal populations contribute to pulmonary fibrosis without evidence for epithelial to mesenchymal transition. Proc. Natl Acad. Sci. USA.

[72] Peng T (2013). Coordination of heart and lung co-development by a multipotent cardiopulmonary progenitor. Nature.

[73] Croft AP (2019). Distinct fibroblast subsets drive inflammation and damage in arthritis. Nature.

[74] Leigh ND (2018). Transcriptomic landscape of the blastema niche in regenerating adult axolotl limbs at single-cell resolution. Nat. Commun..

[75] Gerber, T. et al. Single-cell analysis uncovers convergence of cell identities during axolotl limb regeneration. *Science* **362**, eaaq0681 (2018). 10.1126/science.aaq0681 PMC6669047 30262634

[76] Halpern KB (2018). Paired-cell sequencing enables spatial gene expression mapping of liver endothelial cells. Nat. Biotechnol..

[77] Halpern KB (2017). Single-cell spatial reconstruction reveals global division of labour in the mammalian liver. Nature.

[78] Deutsch A, Feng D, Pessin JE, Shinoda K (2020). The impact of single-cell genomics on adipose tissue research. Int. J. Mol. Sci..

[79] Gupta RK (2012). Zfp423 Expression Identifies Committed Preadipocytes and Localizes to Adipose Endothelial and Perivascular Cells. Cell Metab..

[80] Ferguson GB (2018). Mapping molecular landmarks of human skeletal ontogeny and pluripotent stem cell-derived articular chondrocytes. Nat. Commun..

[81] Reynard LN, Barter MJ (2020). Osteoarthritis year in review 2019: genetics, genomics and epigenetics. Osteoarthr. Cartil..

[82] Vazquez-Liebanas E (2021). Adult-induced genetic ablation distinguishes PDGFB roles in blood-brain barrier maintenance and development. J. Cereb. Blood Flow. Metab..

[83] Armulik A, Genové G, Betsholtz C (2011). Pericytes: developmental, physiological, and pathological perspectives, problems, and promises. Dev. Cell.

[84] Lynch MD, Watt FM (2018). Fibroblast heterogeneity: implications for human disease. J. Clin. Investig..

[85] Ando K (2019). Peri-arterial specification of vascular mural cells from naïve mesenchyme requires Notch signaling. Development.

[86] Cipleu CD, Palant CE, Sanders KM, Dick GM (2002). Separation of two Cl– Currents in Cultured Human and Murine Mesangial Cells: Biophysical and Pharmacological Characteristics of ICl.vol and ICl.Ca. J. Vasc. Res..

[87] Schlondorff D (1987). The glomerular mesangial cell: an expanding role for a specialized pericyte. FASEB J..

[88] Ferland-McCollough D, Slater S, Richard J, Reni C, Mangialardi G (2017). Pericytes, an overlooked player in vascular pathobiology. Pharmacol. Therapeutics.

[89] Leveen P (1994). Mice deficient for PDGF B show renal, cardiovascular, and hematological abnormalities. Genes Dev..

[90] Lindahl P (1998). Paracrine PDGF-B/PDGF-Rβ signaling controls mesangial cell development in kidney glomeruli. Development.

[91] Soriano P (1994). Abnormal kidney development and hematological disorders in PDGF b-receptor mutant mice. Genes Dev..

[92] Kida Y, Duffield JS (2011). Pivotal role of pericytes in kidney fibrosis. Clin. Exp. Pharmacol. Physiol..

[93] Lu Y, Ye Y, Yang Q, Shi S (2017). Single-cell RNA-sequence analysis of mouse glomerular mesangial cells uncovers mesangial cell essential genes. Kidney Int..

[94] Menon R (2018). Single-cell analysis of progenitor cell dynamics and lineage specification in the human fetal kidney. Development.

[95] Karaiskos N (2018). A single-cell transcriptome atlas of the mouse glomerulus. J. Am. Soc. Nephrol..

[96] Kupffer K (1876). Uber Sternzellen der Leber. Briefliche Mitteilung an Professor Waldeyer. Arch. Mikr Anat..

[97] Pinzani M (1995). Hepatic stellate (ITO) cells: expanding roles for a liver-specific pericyte. J. Hepatol..

[98] Geerts A (2001). History, heterogeneity, developmental biology, and functions of quiescent hepatic stellate cells. Semin. Liver Dis..

[99] Mederacke I (2013). Fate tracing reveals hepatic stellate cells as dominant contributors to liver fibrosis independent of its aetiology. Nat. Commun..

[100] Iwakiri Y, Shah V, Rockey DC (2014). Vascular pathobiology in chronic liver disease and cirrhosis—Current status and future directions. J. Hepatol..

[101] Hellstrom M, Kalen M, Lindahl P, Abramsson A, Betsholtz C (1999). Role of PDGF-B and PDGFR-beta in recruitment of vascular smooth muscle cells and pericytes during embryonic blood vessel formation in the mouse. Development.

[102] Dobie R (2019). Single-Cell Transcriptomics Uncovers Zonation of Function in the Mesenchyme during Liver Fibrosis. Cell Rep..

[103] Göritz C (2011). A Pericyte Origin of Spinal Cord Scar Tissue. Science.

[104] Dias DO (2018). Reducing Pericyte-Derived Scarring Promotes Recovery after Spinal Cord Injury. Cell.

[105] Soderblom C (2013). Perivascular Fibroblasts Form the Fibrotic Scar after Contusive Spinal Cord Injury. J. Neurosci..

[106] Dorrier CE (2021). CNS fibroblasts form a fibrotic scar in response to immune cell infiltration. Nat. Neurosci..

[107] Park J (2018). Single-cell transcriptomics of the mouse kidney reveals potential cellular targets of kidney disease. Science.

[108] Papalexi E, Satija R (2018). Single-cell RNA sequencing to explore immune cell heterogeneity. Nat. Rev. Immunol..

[109] Kuppe C (2021). Decoding myofibroblast origins in human kidney fibrosis. Nature.

[110] Xie T (2018). Single-Cell Deconvolution of Fibroblast Heterogeneity in Mouse Pulmonary Fibrosis. Cell Rep..

[111] Kalluri R, Weinberg RA (2009). The basics of epithelial-mesenchymal transition. J. Clin. Investig..

[112] Zhang W (2013). Spatial-temporal targeting of lung-specific mesenchyme by a Tbx4 enhancer. BMC Biol..

[113] Tavassoli M, Crosby WH (1968). Transplantation of marrow to extramedullary sites. Science.

[114] Friedenstein AJ, Chailakhjan RK, Lalykina KS (1970). The development of fibroblast colonies in monolayer cultures of guinea-pig bone marrow and spleen cells. Cell Tissue Kinet..

[115] Frenette PS, Pinho S, Lucas D, Scheiermann C (2013). Mesenchymal stem cell: Keystone of the hematopoietic stem cell niche and a stepping-stone for regenerative medicine. Annu. Rev. Immunol..

[116] Bianco P (2013). The meaning, the sense and the significance: Translating the science of mesenchymal stem cells into medicine. Nat. Med..

[117] Dominici M (2006). Minimal criteria for defining multipotent mesenchymal stromal cells. The International Society for Cellular Therapy position statement. Cytotherapy.

[118] Crisan M (2008). A Perivascular Origin for Mesenchymal Stem Cells in Multiple Human Organs. Cell Stem Cell.

[119] Thomas HM, Cowin AJ, Mills SJ (2017). The importance of pericytes in healing: Wounds and other pathologies. Int. J. Mol. Sci..

[120] Corselli M, Chen CW, Crisan M, Lazzari L, Péault B (2010). Perivascular ancestors of adult multipotent stem cells. Arteriosclerosis, Thrombosis, Vasc. Biol..

[121] Denu RA (2016). Fibroblasts and Mesenchymal Stromal/Stem Cells Are Phenotypically Indistinguishable. Acta Haematologica.

[122] Driskell RR, Watt FM (2015). Understanding fibroblast heterogeneity in the skin. Trends Cell Biol..

[123] Rinkevich Y (2015). Identification and isolation of a dermal lineage with intrinsic fibrogenic potential. Science.

[124] Abbasi S (2020). Distinct Regulatory Programs Control the Latent Regenerative Potential of Dermal Fibroblasts during Wound Healing. Cell Stem Cell.

[125] Soliman H (2020). Pathogenic Potential of Hic1-Expressing Cardiac Stromal Progenitors. Cell Stem Cell.

[126] Joe AWB (2010). Muscle injury activates resident fibro/adipogenic progenitors that facilitate myogenesis. Nat. Cell Biol..

[127] Uezumi A, Fukada SI, Yamamoto N, Takeda S, Tsuchida K (2010). Mesenchymal progenitors distinct from satellite cells contribute to ectopic fat cell formation in skeletal muscle. Nat. Cell Biol..

[128] Pillai ICL (2017). Cardiac Fibroblasts Adopt Osteogenic Fates and Can Be Targeted to Attenuate Pathological Heart Calcification. Cell Stem Cell.

[129] di Carlo SE, Peduto L (2018). The perivascular origin of pathological fibroblasts. J. Clin. Investig..

[130] el Agha E (2017). Mesenchymal Stem Cells in Fibrotic Disease. Cell Stem Cell.

[131] Kramann R, Wongboonsin J, Chang-Panesso M, Machado FG, Humphreys BD (2017). Gli1+ pericyte loss induces capillary rarefaction and proximal tubular injury. J. Am. Soc. Nephrol..

[132] Kramann R (2015). Perivascular Gli1+ progenitors are key contributors to injury-induced organ fibrosis. Cell Stem Cell.

[133] Schneider RK (2017). Gli1+ Mesenchymal Stromal Cells Are a Key Driver of Bone Marrow Fibrosis and an Important Cellular Therapeutic Target. Cell Stem Cell.

[134] Álvarez-Aznar, A. et al. Tamoxifen-independent recombination of reporter genes limits lineage tracing and mosaic analysis using CreERT2 lines. *Transgen. Res.* **29**, 53–68 (2020). 10.1007/s11248-019-00177-8 PMC7000517 31641921

[135] Adams TS (2020). Single-cell RNA-seq reveals ectopic and aberrant lung-resident cell populations in idiopathic pulmonary fibrosis. Sci. Adv..

[136] McLellan MA (2020). High-Resolution Transcriptomic Profiling of the Heart during Chronic Stress Reveals Cellular Drivers of Cardiac Fibrosis and Hypertrophy. Circulation.

[137] Sahai E (2020). A framework for advancing our understanding of cancer- associated fibroblasts. Nat. Rev. Cancer.

[138] Paulsson J, Micke P (2014). Prognostic relevance of cancer-associated fibroblasts in human cancer. Semin. Cancer Biol..

[139] Karnoub AE (2007). Mesenchymal stem cells within tumour stroma promote breast cancer metastasis. Nature.

[140] Raz Y (2018). Bone marrow-derived fibroblasts are a functionally distinct stromal cell population in breast cancer. J. Exp. Med..

[141] Zhang Y (2009). White adipose tissue cells are recruited by experimental tumors and promote cancer progression in mouse models. Cancer Res..

[142] Dirat B (2011). Cancer-associated adipocytes exhibit an activated phenotype and contribute to breast cancer invasion. Cancer Res..

[143] Nieman KM (2011). Adipocytes promote ovarian cancer metastasis and provide energy for rapid tumor growth. Nat. Med..

[144] Lodato MA (2015). Somatic mutation in single human neurons tracks developmental and transcriptional history. Science.

[145] Leung ML (2017). Single-cell DNA sequencing reveals a late-dissemination model in metastatic colorectal cancer. Genome Res..

[146] Kester, L. & van Oudenaarden, A. Single-Cell Transcriptomics Meets Lineage Tracing. *Cell Stem Cell* 1–14 10.1016/j.stem.2018.04.014 (2018). 10.1016/j.stem.2018.04.014 29754780

[147] VanHorn S, Morris SA (2020). Next-Generation Lineage Tracing and Fate Mapping to Interrogate Development. Dev. Cell.

[148] Ziegenhain C (2017). Comparative Analysis of Single-Cell RNA Sequencing Methods. Mol. Cell.

[149] Hagemann-Jensen M (2020). Single-cell RNA counting at allele and isoform resolution using Smart-seq3. Nat. Biotechnol..

[150] Koenitzer JR, Wu H, Atkinson JJ, Brody SL, Humphreys BD (2020). Single-nucleus RNA-sequencing profiling of mouse lung reduced dissociation bias and improved rare cell-type detection compared with single-cell RNA sequencing. Am. J. Respir. Cell Mol. Biol..

[151] Hagberg CE (2018). Flow Cytometry of Mouse and Human Adipocytes for the Analysis of Browning and Cellular Heterogeneity. Cell Rep..

[152] Österreicher CH (2011). Fibroblast-specific protein 1 identifies an inflammatory subpopulation of macrophages in the liver. Proc. Natl Acad. Sci..

[153] Delorey TM (2021). COVID-19 tissue atlases reveal SARS-CoV-2 pathology and cellular targets. Nature.

[154] He S (2020). Single-cell transcriptome profiling an adult human cell atlas of 15 major organs. Genome Biol..

[155] Acosta JR (2017). Single cell transcriptomics suggest that human adipocyte progenitor cells constitute a homogeneous cell population. Stem Cell Res. Ther..

[156] Burl RB (2018). Deconstructing Adipogenesis Induced by β3-Adrenergic Receptor Activation with Single-Cell Expression Profiling. Cell Metab..

[157] Schwalie PC (2018). A stromal cell population that inhibits adipogenesis in mammalian fat depots. Nature.

[158] Vijay J (2020). Single-cell analysis of human adipose tissue identifies depot- and disease-specific cell types. Nat. Metab..

[159] Zhang Z (2019). Dermal adipose tissue has high plasticity and undergoes reversible dedifferentiation in mice. J. Clin. Investig..

[160] Chen Z (2020). Single-cell RNA sequencing highlights the role of inflammatory cancer-associated fibroblasts in bladder urothelial carcinoma. Nat. Commun..

[161] Gao Y (2020). CD63+ Cancer‐Associated Fibroblasts Confer Tamoxifen Resistance to Breast Cancer Cells through Exosomal miR‐22. Adv. Sci..

[162] Wu SZ (2020). Stromal cell diversity associated with immune evasion in human triple‐negative breast cancer. EMBO J..

[163] Dani N (2021). A cellular and spatial map of the choroid plexus across brain ventricles and ages. Cell.

[164] He L (2018). Data Descriptor: Single-cell RNA sequencing of mouse brain and lung vascular and vessel-associated cell types. Sci. Data.

[165] Shi J (2021). Spatio-temporal landscape of mouse epididymal cells and specific mitochondria-rich segments defined by large-scale single-cell RNA-seq. Cell Discov..

[166] de Bakker DEM (2021). Prrx1b restricts fibrosis and promotes Nrg1-dependent cardiomyocyte proliferation during zebrafish heart regeneration. Development.

[167] Farbehi N (2019). Single-cell expression profiling reveals dynamic flux of cardiac stromal, vascular and immune cells in health and injury. eLife.

[168] DeLaughter DM (2016). Single-Cell Resolution of Temporal Gene Expression during Heart Development. Dev. Cell.

[169] Garvin AM (2021). Transient ACE (Angiotensin-Converting Enzyme) Inhibition Suppresses Future Fibrogenic Capacity and Heterogeneity of Cardiac Fibroblast Subpopulations. Hypertension.

[170] Gladka MM (2018). Single-Cell Sequencing of the Healthy and Diseased Heart Reveals Cytoskeleton-Associated Protein 4 as a New Modulator of Fibroblasts Activation. Circulation.

[171] Hulin A (2019). Maturation of heart valve cell populations during postnatal remodeling. Development.

[172] Liang D (2021). Cellular and molecular landscape of mammalian sinoatrial node revealed by single-cell RNA sequencing. Nat. Commun..

[173] Liu Z (2017). Single-cell transcriptomics reconstructs fate conversion from fibroblast to cardiomyocyte. Nature.

[174] Wang L (2021). Single-cell dual-omics reveals the transcriptomic and epigenomic diversity of cardiac non-myocytes. Cardiovasc. Res..

[175] Bahar Halpern K (2020). Lgr5+ telocytes are a signaling source at the intestinal villus tip. Nat. Commun..

[176] Elmentaite R (2020). Single-Cell Sequencing of Developing Human Gut Reveals Transcriptional Links to Childhood Crohn’s Disease. Dev. Cell.

[177] Fawkner-Corbett D (2021). Spatiotemporal analysis of human intestinal development at single-cell resolution. Cell.

[178] Holloway EM (2021). Mapping Development of the Human Intestinal Niche at Single-Cell Resolution. Cell Stem Cell.

[179] Hong SP (2020). Distinct fibroblast subsets regulate lacteal integrity through YAP/TAZ-induced VEGF-C in intestinal villi. Nat. Commun..

[180] Nanus DE (2021). Synovial Tissue from Sites of Joint Pain in Knee Osteoarthritis Patients Exhibits a Differential Phenotype with Distinct Fibroblast Subsets. eBioMedicine.

[181] Kramann R (2018). Parabiosis and single-cell RNA sequencing reveal a limited contribution of monocytes to myofibroblasts in kidney fibrosis. JCI Insight.

[182] Lake BB (2019). A single-nucleus RNA-sequencing pipeline to decipher the molecular anatomy and pathophysiology of human kidneys. Nat. Commun..

[183] Affo S (2021). Promotion of cholangiocarcinoma growth by diverse cancer-associated fibroblast subpopulations. Cancer Cell.

[184] Bhattacharjee S (2021). Tumor restriction by type I collagen opposes tumor-promoting effects of cancer-associated fibroblasts. J. Clin. Investig..

[185] Krenkel O, Hundertmark J, Ritz T, Weiskirchen R, Tacke F (2019). Single Cell RNA Sequencing Identifies Subsets of Hepatic Stellate Cells and Myofibroblasts in Liver Fibrosis. Cells.

[186] Massalha H (2020). A single cell atlas of the human liver tumor microenvironment. Mol. Syst. Biol..

[187] Habermann AC (2020). Single-cell RNA sequencing reveals profibrotic roles of distinct epithelial and mesenchymal lineages in pulmonary fibrosis. Sci. Adv..

[188] Lambrechts D (2018). Phenotype molding of stromal cells in the lung tumor microenvironment. Nat. Med..

[189] Lee JH (2017). Anatomically and Functionally Distinct Lung Mesenchymal Populations Marked by Lgr5 and Lgr6. Cell.

[190] Peyser R (2019). Defining the activated fibroblast population in lung fibrosis using single-cell sequencing. Am. J. Respir. Cell Mol. Biol..

[191] Redente EF (2021). Loss of Fas signaling in fibroblasts impairs homeostatic fibrosis resolution and promotes persistent pulmonary fibrosis. JCI Insight.

[192] Travaglini KJ (2020). A molecular cell atlas of the human lung from single-cell RNA sequencing. Nature.

[193] Valenzi E (2019). Single-cell analysis reveals fibroblast heterogeneity and myofibroblasts in systemic sclerosis-associated interstitial lung disease. Ann. Rheum. Dis..

[194] Wang C (2018). Expansion of hedgehog disrupts mesenchymal identity and induces emphysema phenotype. J. Clin. Investig..

[195] Yun JH (2021). Hedgehog interacting protein-expressing lung fibroblasts suppress lymphocytic inflammation in mice. JCI Insight.

[196] Zepp JA (2017). Distinct Mesenchymal Lineages and Niches Promote Epithelial Self-Renewal and Myofibrogenesis in the Lung. Cell.

[197] Rodda LB (2018). Single-Cell RNA Sequencing of Lymph Node Stromal Cells Reveals Niche-Associated Heterogeneity. Immunity.

[198] Dominguez CX (2020). Single-Cell RNA Sequencing Reveals Stromal Evolution into LRRC15 + Myofibroblasts as a Determinant of Patient Response to Cancer Immunotherapy. Cancer Discov..

[199] Lee JJ (2021). Elucidation of Tumor-Stromal Heterogeneity and the Ligand-Receptor Interactome by Single-Cell Transcriptomics in Real-world Pancreatic Cancer Biopsies. Clin. Cancer Res..

[200] Ligorio M (2019). Stromal Microenvironment Shapes the Intratumoral Architecture of Pancreatic Cancer. Cell.

[201] Lin W (2020). Single-cell transcriptome analysis of tumor and stromal compartments of pancreatic ductal adenocarcinoma primary tumors and metastatic lesions. Genome Med..

[202] Foster DS (2020). Elucidating the fundamental fibrotic processes driving abdominal adhesion formation. Nat. Commun..

[203] Joseph DB (2021). Single-cell analysis of mouse and human prostate reveals novel fibroblasts with specialized distribution and microenvironment interactions. J. Pathol..

[204] de Micheli AJ (2020). Single-cell transcriptomic analysis identifies extensive heterogeneity in the cellular composition of mouse Achilles tendons. Am. J. Physiol. Cell Physiol..

[205] de Micheli AJ (2020). Single-Cell Analysis of the Muscle Stem Cell Hierarchy Identifies Heterotypic Communication Signals Involved in Skeletal Muscle Regeneration. Cell Rep..

[206] Giordani L (2019). High-Dimensional Single-Cell Cartography Reveals Novel Skeletal Muscle-Resident Cell Populations. Mol. Cell.

[207] Harvey T, Flamenco S, Fan CM (2019). A Tppp3 + Pdgfra + tendon stem cell population contributes to regeneration and reveals a shared role for PDGF signalling in regeneration and fibrosis. Nat. Cell Biol..

[208] Johnson GL, Masias EJ, Lehoczky JA (2020). Cellular Heterogeneity and Lineage Restriction during Mouse Digit Tip Regeneration at Single-Cell Resolution. Dev. Cell.

[209] Julien A (2021). Direct contribution of skeletal muscle mesenchymal progenitors to bone repair. Nat. Commun..

[210] Meng S (2020). Reservoir of fibroblasts promotes recovery from limb ischemia. Circulation.

[211] Murach KA (2021). Early satellite cell communication creates a permissive environment for long-term muscle growth. iScience.

[212] Xu Z (2021). Single-cell RNA sequencing and lipidomics reveal cell and lipid dynamics of fat infiltration in skeletal muscle. J. Cachexia, Sarcopenia Muscle.

[213] Deng CC (2021). Single-cell RNA-seq reveals fibroblast heterogeneity and increased mesenchymal fibroblasts in human fibrotic skin diseases. Nat. Commun..

[214] Dobie R (2021). Deciphering mesenchymal drivers of human Dupuytren’s disease at single-cell level. J. Investig. Dermatol..

[215] Guerrero-Juarez CF (2019). Single-cell analysis reveals fibroblast heterogeneity and myeloid-derived adipocyte progenitors in murine skin wounds. Nat. Commun..

[216] He H (2020). Single-cell transcriptome analysis of human skin identifies novel fibroblast subpopulation and enrichment of immune subsets in atopic dermatitis. J. Allergy Clin. Immunol..

[217] Layton TB (2020). Cellular census of human fibrosis defines functionally distinct stromal cell types and states. Nat. Commun..

[218] Leavitt T (2020). Prrx1 Fibroblasts Represent a Pro-fibrotic Lineage in the Mouse Ventral Dermis. Cell Rep..

[219] Mahmoudi S (2019). Heterogeneity in old fibroblasts is linked to variability in reprogramming and wound healing. Nature.

[220] Philippeos C (2018). Spatial and Single-Cell Transcriptional Profiling Identifies Functionally Distinct Human Dermal Fibroblast Subpopulations. J. Investig. Dermatol..

[221] Solé-Boldo L (2020). Single-cell transcriptomes of the human skin reveal age-related loss of fibroblast priming. Commun. Biol..

[222] Tabib T, Morse C, Wang T, Chen W, Lafyatis R (2018). SFRP2/DPP4 and FMO1/LSP1 Define Major Fibroblast Populations in Human Skin. J. Investig. Dermatol..

[223] Tabib T (2021). Myofibroblast transcriptome indicates SFRP2hi fibroblast progenitors in systemic sclerosis skin. Nat. Commun..

[224] Zou Z (2021). A Single-Cell Transcriptomic Atlas of Human Skin Aging. Dev. Cell.

[225] Schiebinger G (2019). Optimal-Transport Analysis of Single-Cell Gene Expression Identifies Developmental Trajectories in Reprogramming. Cell.

