# Supplementary material for: Identification, discrimination and heterogeneity of fibroblasts
Source: Nat Commun. 2022 Jun 14;13:3409. doi: 10.1038/s41467-022-30633-9 (PMC9192344; doi:10.1038/s41467-022-30633-9)
Supplement: Supplementary file 1 — Description of additional supplementary files [file 41467_2022_30633_MOESM1_ESM.docx]

**Legend to Supplementary Data 1.**

A compilation of analyses of fibroblast identity and heterogeneity using scRNA-seq. The list is divided into multi-organ and organ-specific studies, and the latter category is sorted by organ. The species used is also specified. For the list, studies referenced within this review were complemented with studies found from a PubMed® database using the search terms “single-cell RNA sequencing” & “fibroblasts”. Articles from the search result were reviewed and included in the list when they fulfilled the criteria that fibroblasts represented a substantial part of the analysis or were represented in reasonable numbers, such as fibroblasts must represent a substantial part of the analysis. As such, the list should not be considered as complete, but serving as a broad inventory of original studies of fibroblast heterogeneity in different species and organs.
